# Droughts and deforestation: Does seasonality matter?

**DOI:** 10.1371/journal.pone.0276667

**Published:** 2022-10-27

**Authors:** Giuliaz Vaglietti, Philippe Delacote, Antoine Leblois

**Affiliations:** 1 Université de Lorraine, AgroParisTech-INRAE, BETA, Nancy Cedex, France; 2 Climate Economics Chair, Palais Brongniart, Paris, France; 3 CEE-M Univ Montpellier, CNRS INRAE Institut Agro, Montpellier, France; Cranfield University, UNITED KINGDOM

## Abstract

Extreme weather events, particularly droughts, have strong impacts on the livelihoods of populations in rural areas. In a context of low access to insurance and credit markets, households respond to such shocks by implementing different risk-management strategies, which in turn are likely to have an impact on the environment, in particular through land-use changes and deforestation. This paper contributes to the emerging literature on the links between droughts and deforestation: (1) distinguishing responses to previously experienced droughts versus current droughts, and (2) disentangling the time of the agricultural season at which droughts occur. We show that deforestation declines whenever a drought occurs during the growing season, while it increases whenever a drought occurs during the harvesting season. These impacts are mitigated within protected areas and are exacerbated in more accessible locations, i.e., areas within 4 hours of travel time of main/major cities. By contrast, deforestation outcomes following droughts that occur during the planting season depend on whether the crop considered is maize or cassava.

## 1 Introduction

In a context of climate change, shocks linked to meteorological hazards threaten a large number of actors, particularly in countries relying on subsistence rainfed agriculture. In these areas, which are characterized by poor access to credit and insurance markets, a large number of risk-management strategies may be implemented by rural households. Some of these strategies are likely to be related to forest cover change, as they may engender agricultural expansion, forest loss and degradation or, conversely, abandonment of agricultural lands. Such outcomes emphasize the potentially decisive role of meteorological hazards on land use changes.

The literature has recently begun to address the link between droughts and forest loss. Our contribution is two-fold. First, risk-management strategies to face extreme weather events may be implemented either ex-ante or ex-post. Agricultural households adopt ex-ante adaptation strategies based upon their experience of past droughts in an attempt to anticipate future ones. On the other hand, these same households may implement coping strategies when a drought actually occurs (ex-post), as a short-term response to the shock. Our first contribution is to deepen the analysis of household risk-management strategies by comparing how the experience of past and current droughts affect forest loss. Despite only the shock-response strategies having an impact on land-use change are addressed here, the more general terms adaptation and coping are used instead of risk-management. It allows to highlight the differences in the time-response of the strategies: adaptation is used for response induced by past shocks while coping is used for responses induced by current shocks.

Second, the literature addressing deforestation usually deals with annual droughts without looking at the timing of shocks across the agricultural season. However, the timing of rainfall is particularly important. In this article, the effects of experienced and current droughts are broken down according to seasonality, i.e., the time of the agricultural cycle at which a drought occurs.

Our second contribution is to distinguish the impact of droughts on deforestation by analyzing whether they occur during the planting, growing or harvesting season, in a very similar fashion than in Noack *et al*. [1] who address the impact of drought on income. Therefore, our results can be considered complementary to the ones obtained by Noack *et al*. [1] and can be interpreted as the environmental impact of changes in sources of income following a drought. We focus on the Democratic Republic of Congo (DRC), which hosts the world’s second largest tropical forest and where subsistence agriculture is the main cause of forest loss. We consider the country’s two main cultivated crops, i.e., maize and cassava.

In line with other authors, we found that the occurrence of at least one drought in the previous three years induces deforestation reduction. In contrast, current droughts have different effects on same year deforestation, depending on the particular season in which they occur. For instance, the occurrence of drought during the maize planting and growing seasons induces a reduction in deforestation rates while droughts that occur during the harvesting season an increase. Additionally, we investigate how the occurrence of droughts influences the effectiveness of protected areas (PA), which tend to mitigate the impact of droughts on forest loss.

The paper proceeds as follow: Section 2 presents the literature on droughts and deforestation. Section 3 describes our case study, data and identification strategy. Section 4 presents our results. The last section discusses how observed adaptation practices may be related to our results and concludes.

## 2 Literature review: How do droughts impact deforestation?

The impact of degradation and deforestation on climate conditions is well known [2–5], while the opposite mechanism, i.e., the influence of extreme weather events on adaptation strategies involving land-use changes, has been overlooked. The literature explicitly linking extreme weather events, adaptation and land-use change is rather scarce and still emerging. Yet as discussed in Girard et al. [6], adaptation practices related to weather shocks are likely to have an impact on land use.

Some theoretical work has been done to analyze how weather events (and other types of shocks) may impact deforestation. Focusing on non-timber forest products (NTFP) as a safety net against agricultural risk, two mechanisms can be identified. In the long run, Delacote [7] shows that increased risk can lead to lower deforestation. Indeed, if agriculture becomes riskier, land holders may decrease the share of agriculture in their activity portfolio, which leads to lower agricultural expansion and deforestation. In the shorter run, Delacote [8] shows that greater agricultural risk may lead to increased labor allocation to NTFP, which can lead to further forest degradation. In turn, forests biome quality and richness have an essential role in income stabilization, as proven by Noack *et al*. [1]. These papers are part of a larger literature identifying the safety-net role of common property resources first investigated by Baland & Francois [9] and empirically assessed through an experiment in Brunette et al. [10].

**Fig 1:**
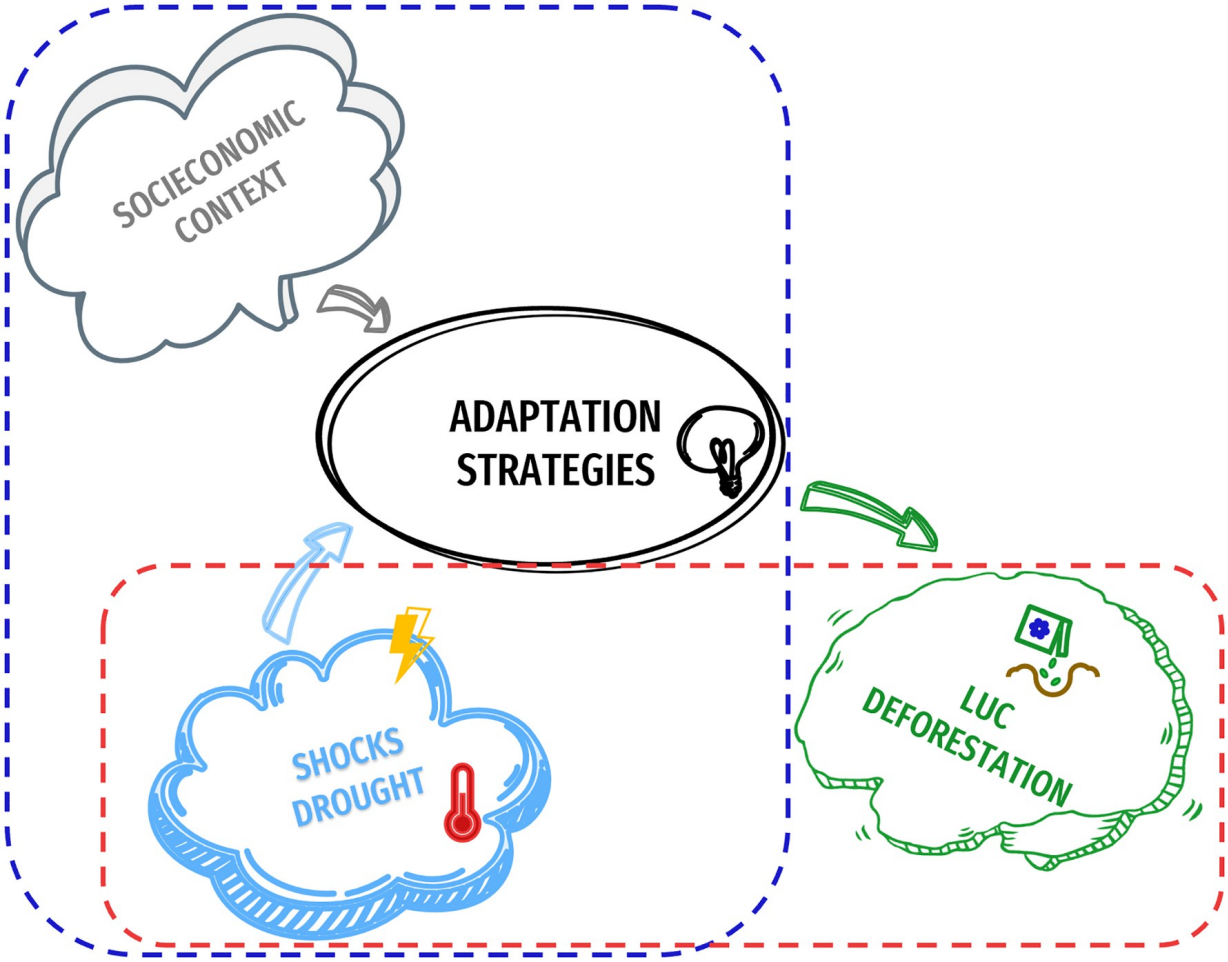
The two sets of empirical literature on droughts, adaptation and land-use change.

Two empirical strategies to approach such issues co-exist and involve data challenges. On the one hand, a branch of the literature (blue box in Fig 1) uses data from households surveys and connects weather shocks with adaptation strategies. Some strategies may be related to land-use changes, but the impact on deforestation may not be assessed, mainly because of a lack of proper spatialization of the data. In this literature, it is shown that the occurrence of drought may lead to: mixed-farming systems [11, 12]; migration with implications both in land use of departure and destination [13, 14]; field relocation and spatial diversification [15–17]; and land expansion [18].

[19] evaluates the effects on forest cover of the adaptation strategies of farmers based on their perception of climate variability. The author found that diversification through off-farm jobs led to forest conservation. In contrast, cattle ranching was identified as a deforestation-driving activity. Eventually migration and pooling were found not to be statistically significantly related to deforestation. The choice of the pursued strategy was eventually found to be connected to the perceived cash benefit derived from forests, as well as to the households’ proximity to commercial and administrative centers: the higher the perceived benefit from NTFP, the higher the willingness of households to preserve the forest cover. Overall, the literature tends to show that adaptation practices implemented under weather shocks are diverse and quite context-dependent.

The second literature branch, which consists of only a few papers, directly deals with the deforestation feedback of weather shocks, relying on remote sensing data (red box of Fig 1). While these data allow for a fine assessment of the relationship between shocks and deforestation, they lack household data on the precise adaptation strategies implemented. Desbureau & Damania [20] study the relationship between droughts and deforestation in Madagascar. They found that the intensity of a drought may lead to divergent adaptation choices and, consequently, may have opposite effects on deforestation. The authors highlight that a mild or moderate drought tends to push farmers to expand agricultural land, which triggers deforestation. More severe droughts push farmers to rely less on agricultural production, which reduces deforestation.

Following a shock, the relationship between rainfall variations and deforestation may be driven by the expansion of plantations. Ruf et al. [21] did not find a significant relationship between droughts, relocation of cocoa plantations and deforestation in Ivory Coast. Alternatively, Zaveri et al. [22], who addressed a similar issue in multiple developing countries, found that dry anomalies caused around 9% of the rate of cropland expansion from 2000 to 2013 and 15% of the deforestation. In the same vein, Staal et al. [23] found that for every mm of water deficit in the Amazon, deforestation tends to increase by 0.13%.

In the case of African rainforest, Gou et al. [24] showed the negative correlation between monthly rainfall amounts and forest disturbances in 2019 and 2020. Most forest disturbance activities indeed occur during the driest months of the year. Focusing on Western and Central Africa, Leblois [25] shows that poor rainy seasons are related to greater deforestation. Moreover, agricultural land endowment and remoteness influences the magnitude of droughts impacts. When the forest cover is large, a short rainfall season leads to a 15% increase in deforestation. In remote areas, the increase in deforestation reaches 20% when the proportion of crop area is small. He & Chen [26] found that extreme heat leads to increases in land holdings and cropland and reduces the forest area. In Ethiopia, only households without enough assets expand cropland, expansion which may substitute for migration and off-farm employment.

Our paper contributes to this second branch of the literature by investigating: 1/ how experienced and current droughts may have diverse effects on deforestation; 2/ how seasonality, that is, the time of the agricultural cycle at which droughts occur, influences the impact on deforestation; 3/ how conservation policies (here protected areas) and distance to cities influence these relationships.

## 3 Droughts and deforestation in the Democratic Republic of Congo

In this section, we first present some contextual issues related to the choice of this case study. Next, we briefly describe the data before presenting the identification strategy.

### 3.1 Case study

Among the countries of the Congo River Basin, which include Cameroon, the Central African Republic, the Republic of Congo, Equatorial Guinea and Gabon, the Democratic Republic of Congo retains the highest share of dense forest, for a total of approximately 155.500.000 ha [27, 28]. Forest accounts for about 55% to 67% of the national territory, depending on the definition of forest adopted. The typologies of forest vary throughout the country, depending on the different agro-climatic zones that the country encompasses. The most common forest typologies are: dense rainforest, swampy forest, dry forest and forest–savannah mosaic [28]. The presence of these various biomes is conditioned by the high diversity of climates in the country. Mean annual precipitations fluctuate around 1,500 mm per year but vary consistently by zone. For example, along the west coast annual cumulative rainfall is only 800 mm, while near the Equator it reaches 2,000 mm. The spatial variability is coupled with a strong inter-annual variability: the nethermost areas are characterized by two rainy seasons (March to May and September to December), while the southeastern area has a single rainy season (July to August) [29]. As shown in the data section, these seasons are reflected in the agricultural seasonality cycle [30].

### 3.2 Deforestation in DRC

Most studies addressing the deforestation dynamics in the DRC are generally part of a wider analysis which focuses on the entire perimeter of the Congo River basin. Among others, the report on deforestation trends by the World Bank [27] suggests the importance of an exclusive focus on the case of the DRC: the report highlights that while deforestation trends in the tropical forests of the DRC are still lower than those observed in neighboring countries, forest degradation is increasing. This may be due to the peculiarities of the country, where sociopolitical instability has prevented the installation of a widespread agriculture and logging industry. In fact, despite being the country with the largest share of biomass and forested area, the DRC has the lowest level of logging activity [27] in in the region. Indeed Fuller et al. [31] assessed the impacts of China’s wood commodity imports from the Congo basin finding the trade from DRC to be negligible with respect to trade with its neighboring countries. This result highlights the fact that forest loss is driven primarily by slash-and-burn agriculture rather than by the commercial timber harvest. Similarly, Tyukavina et al. [32] found small-scale clearing the most dominant form of deforestation: small households were accountable for more than 90% of the total DRC forest cover loss.

Unfortunately, despite the DRC’s peculiarities in terms of deforestation drivers and rates, which make the country an interesting case study, the literature addressing the DRC’s deforestation factors is rather limited. Achille et al. [33] investigated the DRC’s dense forest clearing and degradation dynamics between 1990 and 2018. The authors showed that the total net deforestation rate was around 2.12%, while degradation was 0.12%, with a substantial acceleration after 2005. The authors identified four main deforestation drivers in particular: 1) the population’s heavy dependence on wood for fuel; 2) the practice of itinerant agriculture 3) the presence of mining quarries (also addressed by Davis et al. [34] and Butsic et al. [35]); 4) poor regulatory capabilities in the enforcement of land use rights and land protection. Results are in line with Molinario et al. [36], who investigated the period between 2000 and 2015. The drivers identified by Achille et al. [33] are also potentially connected to the sociopolitical instability, which is recognized as having significant effects on deforestation in developing countries [37]. For example, Butsic et al. [35] found that conflicts in the DRC are a significant driver of deforestation. The authors also found protected areas effective in preventing land clearing, even in the presence of conflicts.

In the DRC, deforestation and degradation are mainly driven by small-scale, subsistence and mostly rainfed agriculture. Given the significant weather variability in the country, addressing how weather shocks, adaptation strategies and forests are connected is of crucial importance, especially considering the fundamental insurance role played by forest biomes in the Congo basin [38]. As mentioned before, the studies addressing the links between deforestation and weather shocks are even more limited, and none have focused on the DRC. For example, while studying the effect of the poor rainy season in Western and Central Africa, Leblois [25] also considers a small portion of the north-western DRC’s tropical forests. Similarly, other global studies, such as Zaveri et al. [22], address the issue of rainfall anomalies in developing countries but do not examine local dynamics and socio-economic factors.

### 3.3 Data description

To investigate the effects of droughts on deforestation, we use three main georeferenced data sets. Final data, information on how these were retrieved and processed, as well as codes for the econometric analysis are available in the data repository supporting this publication [39].

First, the Global Forest Change 2000–2020 database by Hansen et al. [40] offers the fundamental information used to define our dependent variable:

a. the percentage (0–100%) of tree canopy cover for the year 2000 per output grid cell, which represents the canopy closure for all vegetation greater than 5m in height. This variable was provided as a georeferenced database composed by 1 arc second gridded pixel cells (i.e., measuring approximately 30 by 30 meters at the Equator). It was then reshaped to meet the definition of the precipitation database (0.05 decimal degree, around 30km^2^ at the Equator, set as a standard for our unit of the present analysis).
b. a mask layer indicating the state of a cell between mapped pixels, waters or unmapped areas. The mask layer was also reshaped to obtain the total mapped ha for each cell.
c. the year of gross forest cover loss during the period 2000–2020, built as a dummy for each cell indicating whether it was deforested and in which year. A cell is considered as deforested by Hansen et al. [40] only when a clear forest cover cut is observed, i.e. the cell is characterized by a ∼0% crown cover replacement. This selection isolates forest loss attributable to human activities while excluding canopy disturbances.
  Through the aggregation procedure to meet the standard resolution set for the present analysis, this variable was reshaped and transformed to obtain the percentage of each cell deforested every year. We then calculated the total forested area lost for each year in each pixel, through the combination of the data on forest cover loss and the mask layer on mapped areas (point b). The lost area, expressed in hectares, correspond to the final dependent variable applied in the analysis.

**Fig 2:**
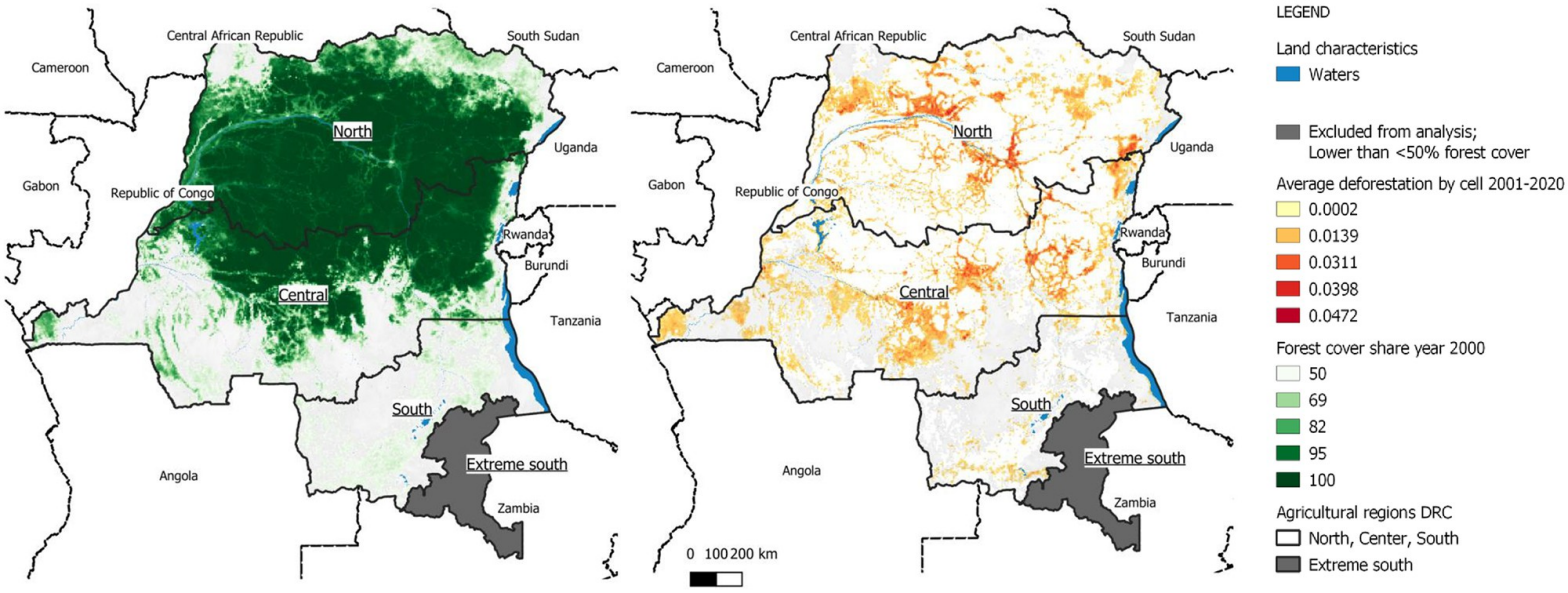
Forest cover in the year 2000, average deforestation 2001–2020, by cell and by agricultural region. Source: GADM [49], Hansen/UMD/Google/USGS/NASA [40] and Crop Calendar Dataset [30].

Forest cover in the year 2000 and the average yearly loss, by pixel, are represented in Fig 2.

A second essential data set is the Crop Calendar proposed by Sacks et al. [30], which provides the georeferenced agricultural calendar for multiple crops. It allows us to divide the country into homogeneous areas of production based on the crop’s agricultural cycle and compute the duration of the planting, growing and harvesting season for each cell and crop.

Due to the importance of maize and cassava in the local diet (respectively 50% and 33% of the total daily intake), [41] these two crops were selected to define the cropping-homogeneous regions of the DRC. Eventually we distinguish four regions (north, center, south and extreme south), each of which is characterized by a different cropping calendar (see data paper).

The cassava planting season extends from April to August in the north, and from October to February in the center, south and extreme south regions and has a single cropping cycle per year. Maize, on the other hand, is characterized by a double cropping cycle per year, so that in the north the planting seasons are from February to March and from June to July. A notable exception is the extreme south region (corresponding to the Haut-Katanga province), which is characterized by a single cropping season even for maize. For this reason, this region was excluded from the analysis.

The duration of seasons in each region was used to estimate a seasonal drought index, based on the average Standardized Precipitation Index (SPI) [42]. The SPI is a standardized index of precipitation quality which informs how much a rainfall observation at a specific time deviates from its long term mean. The index assumes the value of zero whenever the observed rainfalls are around the mean, a positive value (up to +3) for positive deviations, (i.e., a surplus in rainfall) and a negative value (up to -3) when there is a deficit in rainfall. Commonly, the SPI threshold to consider a deficit as a drought is -1. The SPI was calculated on the basis of the monthly precipitation (retrieved from CHIRPS [43]). The drought intensity associated with each grid cell was thus obtained by averaging the monthly SPI for each crop season. Finally, a synthetic drought index was developed, taking value equal to 1 whenever the seasonal average SPI was falling below minus one, and 0 otherwise (dummy variable). A similar approach to seasonality was undertaken by Noack *et al*. [1]. In particular, the authors used the agricultural calendar to quantify the shares of households’ income generated in each season and thereafter the effect of seasonal droughts on income. Nevertheless, the drought index the authors applied is computed as a continuous variable expressing water endowments anomalies associated to the income periods.

Two supplementary variables were added to investigate potential heterogeneity sources. To assess the effectiveness of public policies that addressed forest protection, we used the World Database on Protected Areas (WDPA by UNEP-WCMC [44]). The database provides georeferenced polygons delimiting any protected areas, as well as information about their status, including the year in which the protection was put in place. We then associated a fixed dummy variable to each cell to indicate whether or not the observation was included in a protected area in the year 2000. Lastly, the proximity to cities was retrieved from the Travel Time to Major Cities database [45], which provides for each cell the estimated time required in the year 2000 to reach the nearest city of at least 50,000 inhabitants. Lastly, a dummy variable was created to control for proximity indicating whether a cell was closer than four hours of travelling from a city.

From the processing and merging of the above-mentioned data, we obtained a disaggregated panel database that was constituted of grid cells of approximately 30km^2^ at the Equator. Due to the peculiarity of the original Global Forest Change database, the present analysis covers the years from 2001 through 2020. We use as a benchmark the original state of the forest and the enforced protected areas in the year 2000 (pre-analysis levels) in order to prevent our assessment from potential reverse causality. The final panel database was filtered to maintain only cells that had on average a 50% forest cover in the year 2000: the choice, which allows to retain medium to high forest cover areas, is based on similar thresholds applied by Achard et al. [46, 47] and Verhegghen et al. [48]. For computation matters, the panel was then further randomly sampled to maintain only 35% of the observations, corresponding to 25,958 pixels observed over 20 years (2001–2020).

### 3.4 Descriptive statistics

In the year 2000, the average forest cover of each observation was 86%. Our analysis focuses on forested areas: as highlighted in the data section (3.3), we consider a cell as forested if it had at least 50% of cover in the year 2000. Between 2001 and 2020, in each of these cells, an average of 13 hectares was lost every year (about 0.43% of their area). This result is consistent with results obtained by multiple authors. Achille et al. [33] found a yearly deforestation rate of 0.42% between 1990 and 2018. Our indicator is slightly higher than the average deforestation rate observed by other authors which instead spans from 0.15% to 0.4% in function of the years considered (results resumed by Kengoum et al. [28]). This gap should not surprise, as it can be explained by the increasing trend already observed by Achille et al. [33] and suggested by the World Bank [27] or simply by the particular definition of forests used by each author.

The average cumulative annual rainfall in the DRC reaches 1,692 mm. However, the very large variability in the descriptive statistics (S1 Table in S1 Appendix) can be explained by the country’s wide extension, which defines both the spatial heterogeneity in climate and the observed seasonal intra-annual variations over crop-specific agricultural calendars. For instance, the cumulative rainfall over the cassava agricultural cycle (one per year) ranges from 849 mm during the planting season to 844 mm during the harvesting season. For what concerns the maize’s two annual agricultural cycles, the average cumulative rainfall during the planting seasons is 319 mm (with the first season receiving an average of 100 mm of rain more than than the second). During the growing and harvesting seasons, precipitation is approximately 740 mm and 400 mm, respectively.

The observed cells, which covers the entire country with the exclusion of the Haut-Katanga province, are located at an average travel distance of nine hours and 43 minutes from the nearest main city.

### 3.5 Identification strategy

As mentioned earlier, we distinguish 1/ current and experienced droughts; and 2/ the timing of the drought, to assess how they relate to deforestation.

On the one hand, responses to current droughts are expected to have short-term effects related to coping strategies that may be implemented once the shock occurs. On the other hand, the experience of one or multiple droughts in past years is expected to induce long-term effects related to the outcomes of adaptation strategies.

Our dependent variable *L*_*it*_ is the number of hectares of forest lost in cell *i* at year *t*. The explanatory variables are dummies indicating whether or not there has been a drought during any of the cassava or maize agricultural seasons. A season is considered to be affected by a drought whenever the average monthly SPI falls below -1: in that eventuality, the explanatory variable assumes value equal to 1 and zero otherwise. An important factor is that while the cassava agricultural calendar is composed of a single cycle and only two seasons (planting and harvesting), maize is characterized by two cycles per year (planting/growing/harvesting). Thus, two planting, two growing and two harvesting seasons are observed each year.

In our analysis, both maize cycles are considered jointly. The dummies take the value 1 independently from the cycle that has been hit by drought; that is, if in year *t* a drought hits the cell *i* only in the maize planting season of the first agricultural cycle, then variable *Droughts*_*Maize*_*PL*1, 2_*it*_ takes the value of 1. Similarly, if both the first and second planting seasons are hit by drought, then variable *Droughts*_*Maize*_*PL*1, 2_*it*_ also takes the value of 1.

In order to estimate these impacts, a fixed-effect panel linear regression model is applied. Short-term effects and seasonality are investigated through the following Eq (1):

$$\begin{array}{c} \begin{array}{c} Log\left(L_{\text{it}}+1\right)=\beta_{0}+\beta_{1}Droughts\_Cassava\_PL_{\text{it}}+\beta_{2}Droughts\_Cassava\_HA_{\text{it}}\\ +\beta_{3}Droughts\_Maize\_PL1,2_{\text{it}}+\beta_{4}Droughts\_Maize\_GR1,2_{\text{it}}\\ +\beta_{5}Droughts\_Maize\_HA1,2_{\text{it}}+\gamma_{i}+\delta_{t}+u_{\text{it}} \end{array} \end{array}$$ (1)

The same methodology applies when studying the effects of experienced droughts on current deforestation (Eq (2)). The effect of experienced droughts, as in the aim of model (2), is considered as the effect of any drought that occurred in the 3 years prior to the observed deforestation. Again, the dummy is equal to 1 whenever at least one drought has occurred during any of the two agricultural cycles in the three previous years.

$$\begin{array}{c} \begin{array}{c} Log\left(L_{\text{it}}+1\right)=\beta_{0}+\beta_{1}Exp\_Droughts\_Cassava\_PL_{\text{it}}+\beta_{2}Exp\_Droughts\_Cassava\_HA_{\text{it}}\\ +\beta_{3Exp\_}Droughts\_Maize\_PL1,2_{\text{it}}+\beta_{4}Exp\_Droughts\_Maize\_GR1,2_{\text{it}}\\ +\beta_{5}Exp\_Droughts\_Maize\_HA1,2_{\text{it}}+\gamma_{i}+\delta_{t}+u_{\text{it}} \end{array} \end{array}$$ (2)

In both the models (1) and (2), *γ*_*i*_, *δ*_*t*_, and *u*_*it*_ represent the cell fixed effect, the time fixed effect and the error term, respectively. Standard errors are clustered at the sector level [50], corresponding circa to level 3 of the Global Administrative Area Database (which includes provinces, districts, territories and sectors) [49].

In S2 Appendix, the same models are further developed to better investigate the effect of droughts in each of the two agricultural cycle of maize. Thus, we developed an explanatory variables, again constructed as a dummy, for each of the six maize agricultural seasons observed over a year (first cycle plant, growing, harvesting and second cycle plant, growing, harvesting).

The model of Eq (1) is further developed to investigate the sources of heterogeneity. In particular, we investigate:

a. the role of protected areas. The relationship was estimated by interacting the explanatory variable with a dummy indicating the presence of a PA. More precisely, whenever deforestation was taking place in an area that was protected in the year 2000 (corresponding to the year prior to the beginning of our analysis);
b. the role played by the proximity to cities. The relationship was estimated by interacting the explanatory variable with a dummy indicating whether the deforested cell is at a travel distance of less than four hours from a main city (i.e., a city is considered to be of interest when registering more than 50,000 inhabitants in the year 2000);
c. the role played by the baseline forest cover. Two approaches are undertaken: First by repeating the models presented in Eqs (1) and (2) but maintaining respectively only observations characterized by low forest cover (i.e., minor or equal than 75% in year 2000) or by high forest cover (i.e., higher than 75% in year 2000); Second by interacting both the models with a dummy indicating observations with high forest cover (i.e., assuming value equal to 1 whenever the baseline forest cover is higher than 75%).

Lastly three main robustness checks are undertaken by:

a. converting the dependent variable using an inverse hyperbolic sine transformation of the forest loss, that allows us to retain the multiple zeros present in our database (Summary statistics available in the S1 Appendix). Therefore, the dependent variable of both (1) and (2) is substituted by *arcsinh*(*L*_*it*_) while the explanatory variables remain unchanged;
b. reducing models presented in Eqs (1) and (2) to individually study cassava and maize cycles;
c. excluding from the panel years affected by the Second Congo War.

## 4 Results and discussion

### 4.1 Seasonality matters

The main purpose of this paper is to investigate the effects of droughts on deforestation; first, discerning between experienced and current droughts, and second, investigating the role of seasonality. Again, by seasonality, we mean a period of the agricultural cycle—either planting, growing or harvesting. Recalling that the cassava agricultural cycle is composed of only two seasons, planting and harvesting, is of upmost importance. This peculiarity is due to the reproduction methodology: as cassava is diffused by stems obtained by the plants cropped in the prior agricultural season, and not by seeds, the growing period is less observable with respect to other crops where germination time is required.

#### 4.1.1 Experienced droughts

Experienced droughts over the entire agricultural period do not appear to influence deforestation (S2 Table, column in S2 Appendix). Yet distinguishing the seasons allows us to sharpen the analysis. Table 1, column (1) illustrates the effects of experienced droughts, i.e., those that occurred during the three years before the observed deforestation. While previous shocks during the cassava agricultural cycle do not seem to induce any deforestation, experienced droughts hitting the maize growing or harvesting season tend to decrease deforestation in the following years. More precisely, droughts that occurred during the maize growing season reduce current deforestation by 2.6%, i.e., of 0.33 hectares less than the average 12.96 hectares lost on average in each cell; while they reduce it during the harvesting season by 1.9%. The overall consequence of experienced droughts is reduced deforestation. This result suggests that household adaptation strategies may imply a reallocation of the activity portfolio from agriculture to off-farm activities, including a higher reliance on NTFP harvesting.

**Table 1:** Deforestation in response to experienced and current droughts. Analysis of cells characterized by at least 50% forest cover in the year 2000. The observations are then sampled to maintain the 35% of their total.

	*Dependent variable*:	
	Log of deforested hectares + 1	
	(1) Experienced Droughts	(2) Current Droughts
Cassava		
Planting	−0.0186 (0.0248)	0.0569** (0.0249)
Harvesting	−0.0597 (0.0441)	−0.0636 (0.0535)
Maize		
Planting 1,2	0.0150 (0.0172)	−0.0304** (0.0136)
Growing 1,2	−0.0256* (0.0150)	−0.0689*** (0.0199)
Harvesting 1,2	−0.0194* (0.0115)	0.0592*** (0.0129)
Observations	519,160	519,160
F Statistic (*df* = 5;493178)	37.6635***	73.8697***

Note:

*p < 0.1;

**p < 0.05;

***p < 0.01

Time and cell fixed effects, clustered at the sector administrative level

These results are in line with the results from Desbureau & Damania [20], who found that repeated droughts in the past tend to decrease deforestation. This outcome was also predicted in a theoretical model by Delacote [7], who suggest that a higher perceived risk in agriculture may lead households to diversify their livelihood strategy, reducing their share of agriculture but relying more on forests, which would lead to a lower deforestation rate. Nevertheless, a household’s higher reliance on NTFP may imply a higher level of forest degradation [8], which has not been investigated in this paper.

Interestingly, when we exclude the war years prior to 2004 to check the robustness of the previously discussed results, results concern experienced droughts that occurred anytime over the agricultural year proved to be significant, confirming that experienced droughts tend to reduce deforestation. These outcomes suggest that there is room for further research investigating the interactions between conflicts, droughts and deforestation.

#### 4.1.2 Current droughts

When we look at the occurrences of current droughts over the entire agricultural period, they have no significant influence on deforestation (S2 Table, column in S2 Appendix), but if we distinguish these occurrences by season, they do. Column (2) of Table 1 reports the effects of current droughts affecting the deforestation rate in the same year the shocks are observed. Interestingly, deforestation rates take different directions in function of the agricultural season affected. A shock in the cassava planting or maize harvesting season increases deforestation by 5.7% and 5.9%, respectively, while droughts in the maize planting or growing season decrease forest clearing by 3.0% and 6.9%, respectively.

Focusing on cassava, experienced and current droughts do not have the same link with deforestation. This may be explained by the peculiarity of the crop itself. Cassava has a long agricultural cycle which spans the entire year. Thus, the cycle can be adjusted with respect to the households’ experience of the previous years and to better match previously observed rainfall patterns. Instead, for what it concerns current droughts, a shock during the planting season may induce households in the short term to expand the agricultural area to maintain the same level of production: indeed the first three to four months are the most delicate time of the cycle, when the cassava stem needs a substantial amount of moisture to properly develop the roots [51]. Otherwise cassava can be considered quite resistant to weather shocks [52], which can explain our non-significant results about droughts occurring during the harvesting period.

Focusing on maize, it is more difficult to adjust the timing of the planting and harvesting seasons due to the double cropping system adopted in the DRC. Thus households, when observing a drought at the beginning of the cycle or in the most delicate period of it, the growing season, may immediately reallocate their portfolio, in order to reduce the risk of loss. These results may be compared to those obtained by Leblois [25] in the Guineo-Congolian regions, where the author found that a drought during the rain season reduces deforestation.

The interpretation of increased deforestation during the maize harvesting period is different. Two main contemporaneous mechanisms may be suggested: first, experiencing a drought during a harvesting season may increase households’ expectations of observing a drought in the following planting season. Thus, households may anticipate the shock and clear land during the harvesting period to have a wider area dedicated to agriculture in the following cycle and to thus maintain the level of output. Second, considering that the harvesting period is generally the driest of the cycle, a drought occurring in that season may significantly ease accessibility to forests, reducing the efforts required by clearing. A similar eventuality was also suggested by Leblois [25], in analyzing the effects of a short rainy season on deforestation. The two highlighted mechanisms, working together, may explain greater forest loss when droughts occur during the maize harvesting season.

These results are complementary to the ones proposed by Noack et al. [1]. In the eventuality of droughts during the growing season, the authors observed that the share of rural income coming from forest products was increasing. This result therefore justifies our findings that deforestation decreases in the eventuality of a shock during the growing season, potentially due to a higher opportunity cost of maintaining the forest asset rather than expanding the agricultural area amid the agricultural cycle. In turn, in the eventuality of a shock during the harvesting season, Noack et al. observed an increase in the share of income generated through agriculture while we observe an increase in deforestation. As in rural areas an increase in agricultural income may be reached mainly through the expansion of the cultivated surfaces, Noack et al. support our hypothesis that forest is lost due to agricultural land expansion.

A general takeaway from these results is that inter and intra-annual dynamics are characterized by extremely different responses which also vary depending on the crop grown in the area. In fact, drought responses clearly depend on the crops affected, notably on the type of seasonal growing periods, e.g., double cropping or single cropping. It follows that, in order to effectively protect forests, policy makers should carefully consider the relationship between the vulnerability of local farmers, weather events and deforestation.

### 4.2 Looking for sources of heterogeneity in the land-use response to weather shocks

In this section, we investigate the sources of heterogeneity influencing deforestation, as a response to current droughts. The interactions with the first model are not presented, due to the already low significance of the effect of experienced droughts (Table 1, column 1).

**Fig 3:**
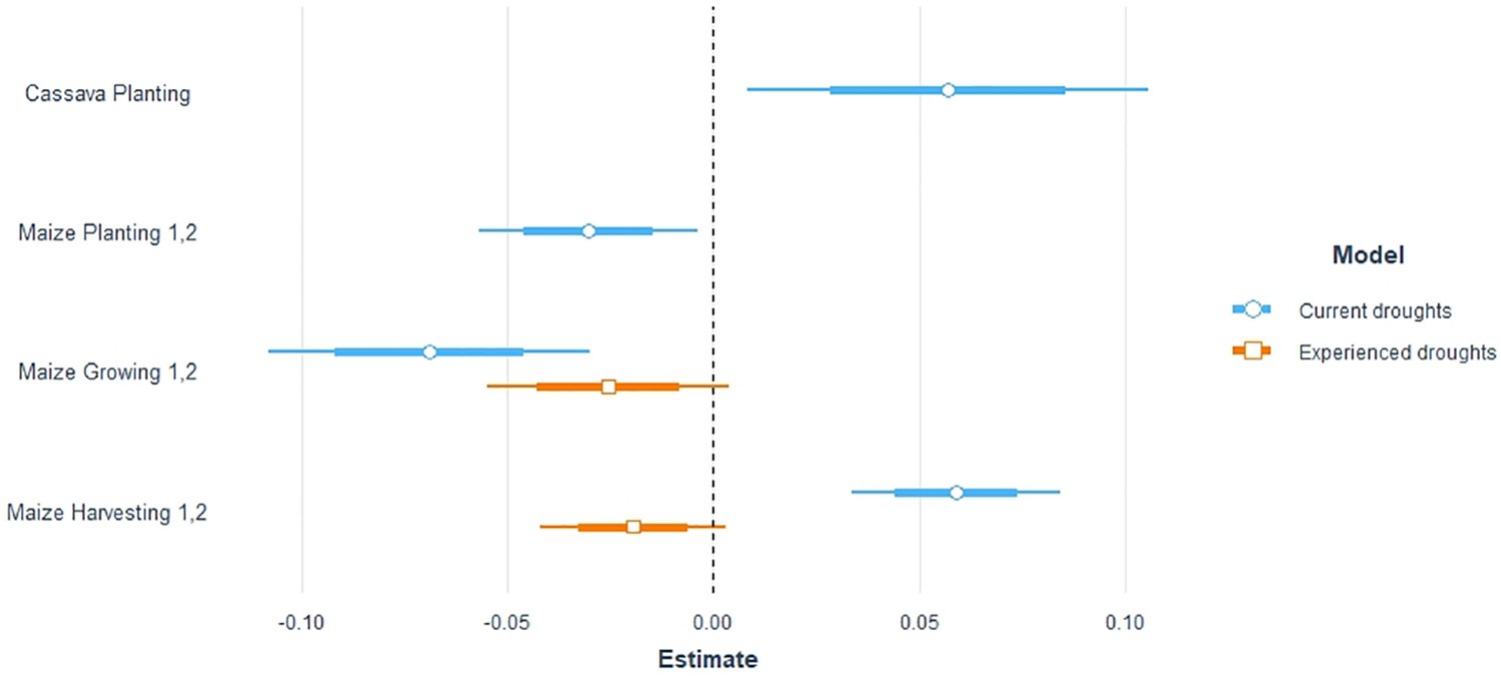
Deforestation and current droughts; graphical representation of deforestation in response to experienced and current droughts.

**Fig 4:**
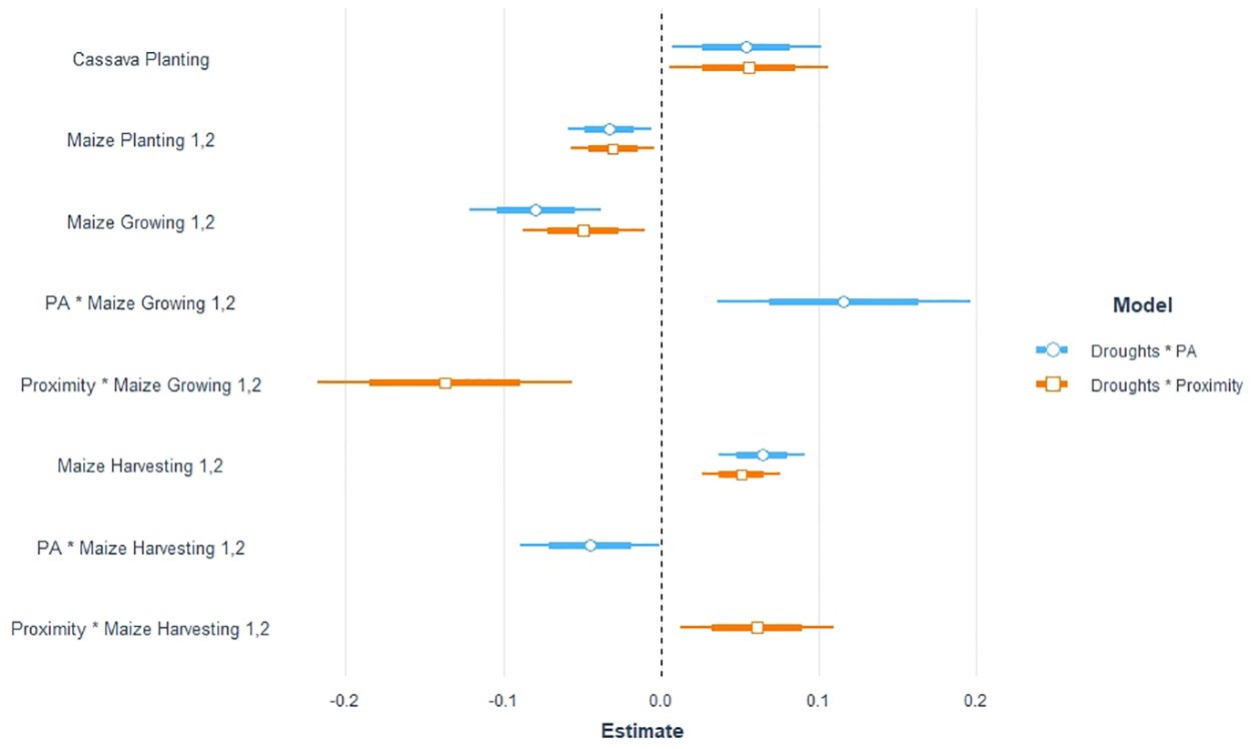
Deforestation and current droughts; graphical representation of interaction with protected areas and proximity to main cities.

In particular, we analyze the influence of conservation policies, economic pressure and forest cover. Conservation policies are represented by protected areas, while economic pressure is measured by proximity to cities, which entails lower transport costs and better outside options for households. The current drought indicators were interacted with these two variables. The main results are presented in Table 2 while the most significant ones are displayed in Figs 3 and 4. The influence of the forest endowment is instead investigated in function of the observed forest cover share in year 2000 (Tables 3 and 4).

**Table 2:** Deforestation and current droughts; interaction with protected areas (installed before the year 2000) and proximity to main cities. The analysis considers cells characterized by least 50% forest cover in the year 2000. Total observations are then sampled to maintain only the 35% of the total.

	*Dependent variable*:	
	Log of deforested hectares + 1	
	(1) Current droughts × Protected Areas	(2) Current droughts × Proximity
Cassava		
Planting	0.0541** (0.0241)	0.0556** (0.0259)
Harvesting	−0.0598 (0.0570)	−0.0487 (0.0532)
Maize		
Planting 1,2	−0.0325**(0.0136)	−0.0306**(0.0137)
Growing 1,2	−0.0796***(0.0214)	−0.0492**(0.0197)
Harvesting 1,2	0.0640***(0.0139)	0.0511***(0.0125)
Interactions:	Protected Areas ×	Proximity ×
× Cassava Planting	0.0115(0.0762)	0.0269(0.0531)
× Cassava Harvesting	−0.0635 (0.1149)	−0.1262 (0.1233)
× Maize Planting 1, 2	0.0230 (0.0485)	-0.0060(0.0282)
× Maize Growing 1, 2	0.1161*** (0.0409)	−0.1371*** (0.0413)
× Maize Harvesting 1, 2	−0.0453** (0.0226)	0.0610** (0.0247)
Observations	519,160	519,160
F Statistic (*df* = 10;493173)	41.6811***	45.8807***

Note:

*p < 0.1;

**p < 0.05;

***p < 0.01

Time and cell fixed effects, clustered at the sector administrative level

**Table 3:** Deforestation and droughts over the main agricultural periods and cycles, differentiating between low (≤ 75%) and high (> 75%) forest cover in year 2000.

	*Dependent variable*:			
	Log of deforested hectares + 1			
	Experienced droughts		Current droughts	
	(1) Low forest cover	(2) High forest cover	(3) Low forest cover	(4) High forest cover
Cassava				
Planting	-0.1245*** (0.0407)	0.0211 (0.0295)	0.0967** (0.0379)	0.0458 (0.0314)
Harvesting	-0.2240** (0.1060)	-0.0020 (0.0346)	-0.1069 (0.1673)	-0.0886** (0.0435)
Maize				
Planting 1,2	0.0634 (0.0391)	-0.0075 (0.0159)	-0.0403 (0.0270)	-0.0196 (0.0133)
Growing 1,2	-0.0083 (0.0234)	-0.0376** (0.0175)	-0.0780** (0.0310)	-0.0678*** (0.0232)
Harvesting 1,2	0.0158 (0.0236)	-0.0306** (0.0119)	0.0749*** (0.0222)	0.0430*** (0.0134)
Observations	141,920	377,240	141,920	377,240
F Statistic	54.2955***	41.4728***	26.6673***	41.3791***
	(df = 5; 134800)	(df = 5; 358354)	(df = 5; 134800)	(df = 5; 358354)

Note:

*p < 0.1;

**p < 0.05;

***p < 0.01

Time and cell fixed effects, clustered at the sector administrative level

**Table 4:** Deforestation and droughts over the main agricultural periods and cycles, interaction with high baseline forest cover. Total observations are then sampled to maintain only the 35% of the total observations.

	*Dependent variable*:	
	Log of deforested hectares + 1	
	(1) Experienced droughts × High forest cover × High forest cover	(2) Current droughts × High forest cover × High forest cover
Cassava		
Planting	-0.1082** (0.0428)	0.0558(0.0362)
Harvesting	-0.2078* (0.1079)	-0.0551 (0.1710)
Maize		
Planting 1,2	0.0600 (0.0371)	-0.0572** (0.0263)
Growing 1,2	0.0040 (0.0243)	-0.0761** (0.0332)
Harvesting 1,2	-0.00001 (0.0219)	0.1023*** (0.0212)
Interactions:	High forest cover ×	High forest cover ×
× Cassava Planting	0.1300** (0.0508)	-0.0023 (0.0435)
× Cassava Harvesting	0.2078* (0.1090)	-0.0121 (0.1695)
× Maize Planting 1, 2	-0.0643* (0.0365)	0.0406 (0.0269)
× Maize Growing 1, 2	-0.0434 (0.0289)	0.0077 (0.0383)
× Maize Harvesting 1, 2	-0.0273 (0.0222)	-0.0599*** (0.0226)
Observations	519,160	519,160
F Statistic (df = 10; 493173)	45.2956***	43.4697***

Note:

*p < 0.1;

**p < 0.05;

***p < 0.01

Time and cell fixed effects, clustered at the sector administrative level

The general seasonality effects that are found in the previous section are robust to the addition of these interactions. Overall, protected areas tend to mitigate some (positive or negative) effects of droughts on deforestation, while proximity to cities tends to exacerbate them.

#### 4.2.1 Impact of protected areas

The interactions between protected areas and seasonal droughts is significant in two seasons: the maize growing and harvesting seasons (Table 2, column 2). In both cases, the presence of protected areas mitigates the overall impact of droughts, whether it is positive or negative.

During the maize harvesting season, the presence of protected areas offsets the increased deforestation (-4.5%) that is generally observed during a drought in the same season but in a non-protected location (+6.4%). This result is in line with Desbureau & Damania [20] who found that, in Madagascar, protected areas mitigate the impact of droughts.

Conversely, the decrease in deforestation when a drought occurs during the maize growing season (-8%) is over compensated for when it takes place in protected areas (11.6%). Even if counter intuitive, this result may be explained by the peculiarities of the location of protected areas as well as by the growing season. In fact, the growing season can be considered more sensitive to droughts, in the sense that, in low input agriculture, it allows for almost no margin of adjustment. Moreover, protected forests are a warranty of high ecosystem services provision (for example NTFP collection) in areas that are generally remote and where reallocation to off-farm/forest jobs is mostly unlikely. A similar result was observed by Noack et al. [1], who highlighted the role of biodiversity in reducing drought shocks on income. Therefore, thanks to this smoothing effect, the opportunity cost to clear forest for cultivation increases. The combination of these two factors may induce greater pressure on forest resources in drought periods, leading to a higher deforestation rate.

#### 4.2.2 Proximity to cities

The variable proximity can be considered as a proxy of accessibility to markets, as well as to off-farm jobs. As for the presence of protected areas, we found a significant impact of the interaction between droughts and proximity to cities, in the case of the maize growing and harvesting seasons (Table 2, model 2). In both cases, proximity to cities exacerbates the (positive or negative) impact of droughts on deforestation.

During the growing season, proximity to cities tends to preserve more forests by reducing land clearing (-13.7%), while the overall effect is positive (+4, 9%). Conversely, in the harvesting season, proximity amplifies deforestation in case of droughts (6.1%), while the general effect is negative.

This magnification of the drought effect further confirms the likelihood of the previously suggested coping strategies: during the growing season, where adjustments to maintain the same level of output are no longer possible and labor is free, better access to off-farm jobs reduces deforestation. Instead, during the harvesting season, the increment in deforestation suggests that proximity to cities may induce households to increase land exploitation in an attempt to maintain the same output level in the subsequent seasons. In this specific context, any eventual production surplus can be placed in a more efficient market with respect to the ones in remote areas. These effects are likely to add up with the deforestation trends of higher income areas, where households demand is generally oriented towards more land-intensive goods and where the demand for agricultural products increases faster than for forest ones [53].

#### 4.2.3 Forest cover

As introduced in Section 3.5, we applied two complementary approaches to estimate the influence of forest cover on droughts impacts. In line with our main analysis, both methods confirm the existence of heterogeneity depending on the time of crop and the timing of shock across the agricultural season, as well as the pixel share of forest cover.

In the first approach, the model assessing current and experienced droughts (Section 3.5 Eq 1 and 2) are applied to two subsets of our total sample, distinguished in function of the observed baseline forest cover: low (≤ 75%) and high (> 75%).

Regarding current droughts in the maize growing and harvesting season, where respectively deforestation decreases and increases, the impacts are smoothed in dense forest (Table 3, column 3 and 4). A difference of -1.02 percentage points is observed when comparing deforestation in the maize growing season in low cover areas (-7.8%) versus high cover ones (-6.7%). Similarly, a difference of -3.19 percentage points between low and high forest cover cells is observed when comparing the deforestation increases following a drought in the harvesting season (respectively +7.4% and +4.3%).

This is potentially due to the limited capacity to adjust in the short term in areas characterised by dense forests where relocation of inputs is only foreseeable in the long term. This hypothesis is confirmed by the strong reduction in deforestation observed in the experienced drought model (Table 3, column column 1 and column 2). In particular, experienced droughts occurring during the cassava agricultural cycle influences more significantly deforestation in low forest cover areas while maize is more influential in high cover ones (Table 3, column 1 and 2).

In second instance, both the models are further interacted with a dummy variable indicating high forest cover (Table 4), in a similar fashion as done in investigating the role of protected areas or proximity to cities (Sections 4.2.1 and 4.2.2).

In line with our main results, experienced droughts tend to decrease deforestation. Nevertheless, for what it concerns the entire agricultural cycle of cassava, is observed an increase in deforestation in areas characterized by high forest cover. These results may reflect the potential different importance of the two crops in function of the forest cover, potentially related to their biological characteristics, and suggesting that in dense forest cassava expansion may be a long term adaptation strategy at the detriment of maize cropping. For what concerns the interaction of forest cover with current droughts, high forest cover tends to mitigate drought impacts in the maize harvesting season, in a manner that is similar to the one observed in protected areas. No significant impact in deforestation is observed in the maize growing season, contrarily o what is observed in protected areas which therefore would require a targeted attention of protection policies (see Section 4.2.1).

### 4.3 Robustness checks

We provide three main robustness checks of the previous models (displayed in S3 Appendix). In the first check, the dependent variable is transformed using an inverse hyperbolic sine transformation. Results, displayed in S3 Table of S3 Appendix are not significantly different than those obtained applying the model of Eq (1). Secondly,the cassava and maize cycles are individually studied. Again, intensity, direction and significance did not remarkably vary from the main models discussed in the results section (S4 and S5 Tables in S3 Appendix). Lastly, the DRC has been shaken by a long period of instability. The Second Congo War took place between 1998 and 2003. It affected the role of the State, compromising its functioning [35, 54], as well as households’ behaviors concerning land use and forest access. For this reason, the Eq (1) is estimated excluding the years of war. Again the significant variables maintain the same intensity and direction as in the previously discussed models (S6 Table in S3 Appendix).

## 5 Conclusion

In a context of climate change, extreme weather events and droughts put many rural populations at risk in the developing world, especially in areas where agriculture is rainfed. Many adaptation strategies can be implemented and depend on socio-economic contexts. Such practices are likely to have environmental impact, especially on land-use change and deforestation.

This paper, which focuses on the Democratic Republic of Congo, contributes to the scarce literature linking land-use changes to droughts. First, we underline the differences between experienced (past) and current droughts with respect to their impact on deforestation: while experienced droughts impact deforestation in several seasons, current droughts influence it in different directions depending on when they occur. The main intuition behind these results is that an experienced drought can be understood as an indicator of future drought expectations by land holders, influencing long-term adaptation. In contrast, current droughts are the variable that land holders consider for implementing their short-term coping strategies.

Second, in the literature addressing deforestation dynamics, droughts impacts are aggregated over the whole agricultural cycle, if not over the entire year. Our results show that seasonality, i.e., the time of the cycle at which the drought takes place, matters. We distinguish between the planting, growing and harvesting seasons of the DRC’s two main cultivated crops: casava and maize. The effect of droughts varies with respect to the crop affected and the structure of its agricultural cycle (single or double cropping). Multiple deforestation outcomes are possible: in the case of maize, a drought in the planting or growing season decreases deforestation, while it tends to increase it in the harvesting season.

Third, some sources of heterogeneity of these impacts are investigated: namely the impact of proximity to cities, of protected areas and of forest cover observed in year 2000, before the beginning of the analysis. Protected areas have been proven to offset the (positive or negative) impact of droughts on deforestation, in two distinct seasons of the maize cycle: in the growing season, the impact of droughts on deforestation is less negative within PAs than outside; in the harvesting season, it is less positive. In contrast, proximity to cities amplifies the effect of a drought on deforestation: in the growing season, droughts reduce deforestation more near cities than further away from them; in the harvesting season, droughts increase deforestation more. Nevertheless, impacts may vary in function of the local forest cover and relative importance of cassava and maize.

In light of these results, it appears that policymakers should take into account the interactions between droughts and coping strategies to be able to prevent undesired deforestation outcomes and simultaneously ensure better livelihood resilience to local populations. These considerations imply a need for even more importance given the increasing interest of the international community in preserving tropical forests, and in particular the Congo Basin. Part of the COP26 incentives were devoted to supporting sound governance and the development of an economic model that aims to support agriculture or energy investments with a reduced impact on forests.

The remote sensing data used in this analysis is highly informative, both on the state of forests and on the intensity of weather events. Yet the local population’s perception of and reactions to shocks are not explicitly considered. In particular, adaptation and coping strategies, including input reallocation, are not directly observed. Our results open questions and challenges for further research in order to link droughts, farmers’ responses to shocks and forest use.

## Supporting information

S1 Appendix Descriptive statistics. (PDF)

S2 Appendix Main results. (PDF)

S3 Appendix Robustness checks. (PDF)

S1 File (HTML)
