# Supplementary material for: Droughts and deforestation: Does seasonality matter?
Source: PLoS One. 2022 Oct 27;17(10):e0276667. doi: 10.1371/journal.pone.0276667 (PMC9612518; doi:10.1371/journal.pone.0276667)
Supplement: S1 Appendix — (PDF) [file pone.0276667.s001.pdf]

A Appendix: Descriptive statistics

S1 Table. Descriptive statistics

Statistic	N	Mean	St. Dev.	Min	Pctl(25)	Pctl(75)	Max
Forest Cover year 2000 (%)	519,160	86.0358	17.5015	50.0032	71.0436	99.6546	99.9996
Rainfall (mm/y)	519,160	1,691.9000	254.1412	629.4420	1,539.2810	1,842.6380	3,074.1600
Lost hectares	519,160	12.9631	29.1887	0	0	11.3	1,614
Travel time to 50k city (min)	519,160	583.9865	406.9662	13.3611	312.8889	729.1389	3,542.4440
Proximity, travel time ¡4h (dummy)	519,160	0.1423	0.3494	0	0	0	1
Rainfalls (mm)							
Maize Planting 1	519,160	373.4552	177.9664	29.9399	232.2017	533.5520	1,109.8310
Maize Planting 2	519,160	264.9211	100.1379	4.3111	194.2088	330.7671	902.5035
Maize Growing 1	519,160	710.6152	153.1911	271.5063	595.0576	820.3250	1,459.5060
Maize Growing 2	519,160	769.2537	139.6965	102.9565	682.9990	837.4545	1,588.2320
Maize Harvesting 1	519,160	338.5848	146.4111	2.1306	254.4054	437.2237	877.7123
Maize Harvesting 2	519,160	456.3089	124.5292	109.3898	365.7688	541.7220	1,109.8310
Cassava Planting	519,160	849.0816	139.1017	232.6096	766.8894	933.0655	1,635.3680
Cassava Harvesting	519,160	844.2288	250.0814	111.9972	669.1016	1,016.7890	1,761.0920
SPI							
Maize Planting 1	519,160	-0.0059	0.5942	-2.9203	-0.3855	0.3886	2.6239
Maize Planting 2	519,160	-0.0508	0.5940	-2.4539	-0.4410	0.3471	2.2145
Maize Growing 1	519,160	-0.0218	0.4530	-1.8037	-0.3290	0.2819	1.9149
Maize Growing 2	519,160	-0.0496	0.4631	-2.3159	-0.3497	0.2514	1.8733
Maize Harvesting 1	519,160	-0.0089	0.5841	-2.3795	-0.3895	0.3810	2.3714
Maize Harvesting 2	519,160	-0.0586	0.6319	-3.0377	-0.4641	0.3518	2.7167
Cassava Planting	519,160	-0.0499	0.4354	-2.2637	-0.3311	0.2303	1.8187
Cassava Harvesting	519,160	-0.0401	0.3596	-1.5003	-0.2808	0.1920	1.4081
Current droughts, dummy							
Maize Planting 1	519,160	0.0506	0.2193	0	0	0	1
Maize Planting 2	519,160	0.0568	0.2314	0	0	0	1
Maize Growing 1	519,160	0.0166	0.1278	0	0	0	1
Maize Growing 2	519,160	0.0211	0.1437	0	0	0	1
Maize Harvesting 1	519,160	0.0491	0.2160	0	0	0	1
Maize Harvesting 2	519,160	0.0647	0.2460	0	0	0	1
Cassava Planting	519,160	0.0175	0.1310	0	0	0	1
Cassava Harvesting	519,160	0.0037	0.0607	0	0	0	1
Maize Planting 1,2	519,160	0.1037	0.3048	0	0	0	1
Maize Growing 1,2	519,160	0.0363	0.1870	0	0	0	1
Maize Harvesting 1,2	519,160	0.1087	0.3112	0	0	0	1
All year	519,160	0.1891	0.3916	0	0	0	1
Experienced droughts, dummy							
Maize Planting 1	519,160	0.1422	0.3492	0	0	0	1
Maize Planting 2	519,160	0.1453	0.3524	0	0	0	1
Maize Growing 1	519,160	0.0453	0.2079	0	0	0	1
Maize Growing 2	519,160	0.0587	0.2351	0	0	0	1
Maize Harvesting 1	519,160	0.1313	0.3377	0	0	0	1
Maize Harvesting 2	519,160	0.1757	0.3806	0	0	0	1
Cassava Planting	519,160	0.0492	0.2162	0	0	0	1
Cassava Harvesting	519,160	0.0099	0.0992	0	0	0	1
Maize Planting 1,2	519,160	0.2672	0.4425	0	0	1	1
Maize Growing 1,2	519,160	0.0985	0.2980	0	0	0	1
Maize Harvesting 1,2	519,160	0.2830	0.4504	0	0	1	1
All year	519,160	0.4172	0.4931	0	0	1	1
