# Supplementary material for: Droughts and deforestation: Does seasonality matter?
Source: PLoS One. 2022 Oct 27;17(10):e0276667. doi: 10.1371/journal.pone.0276667 (PMC9612518; doi:10.1371/journal.pone.0276667)
Supplement: S2 Appendix — (PDF) [file pone.0276667.s002.pdf]

B Appendix: main results

S2 Table.Experienced droughts, current droughts, seasonality. Deforestation and droughts over the main agricultural periods and cycles; experienced and current, yearly aggregation and by season

	Dependent variable:				
	Log of deforested hectares +1				
Droughts:	(1) Experienced & current yearly aggregation	(2) Experienced, detailed	(3) Current, detailed	(4) Current, aggregated	(5) Experienced, aggregated
Experienced, y	-0.0180				
	(0.0115)				
Current, y	-0.0005				
	(0.0110)				
Cassava					
Planting		-0.0182	0.0605^{**}	0.0569^{**}	-0.0186
		(0.0255)	(0.0250)	(0.0249)	(0.0248)
Harvesting		-0.0576	-0.0886^{*}	-0.0636	-0.0597
		(0.0420)	(0.0519)	(0.0535)	(0.0441)
Maize					
Planting 1		0.0094	-0.0674^{***}		
		(0.0172)	(0.0187)		
Planting 2		0.0060	-0.0243		
		(0.0179)	(0.0227)		
Growing 1		-0.0531^{**}	-0.0717^{***}		
		(0.0227)	(0.0275)		
Growing 2		0.0099	-0.0589^{**}		
		(0.0214)	(0.0253)		
Harvesting 1		-0.0352^{*}	0.0915^{***}		
		(0.0192)	(0.0255)		
Harvesting 2		-0.0026	0.0264^{*}		
		(0.0134)	(0.0147)		
Planting 1,2				-0.0304^{**}	0.0150
				(0.0136)	(0.0172)
Growing 1,2				-0.0689^{***}	-0.0256^{*}
				(0.0199)	(0.0150)
Harvesting 1,2				0.0592^{***}	-0.0194^{*}
				(0.0129)	(0.0115)
Observations	519,160	519,160	519,160	519,160	519,160
F Statistic	27.8757^{***}	33.7886^{***}	69.5683^{***}	73.8697^{***}	37.6635^{***}
	(df = 2; 493181)	(df = 8; 493175)	(df = 8; 493175)	(df = 5; 493178)	(df = 5; 493178)
Note:					$^{*}p < 0.1$; $^{**}p < 0.05$; $^{***}p < 0.01$
					Time and cell fixed effects, clustered at the sector administrative level
