# Supplementary material for: Droughts and deforestation: Does seasonality matter?
Source: PLoS One. 2022 Oct 27;17(10):e0276667. doi: 10.1371/journal.pone.0276667 (PMC9612518; doi:10.1371/journal.pone.0276667)
Supplement: S3 Appendix — (PDF) [file pone.0276667.s003.pdf]

C Appendix: Robustness checks

S3 Table. Dependent variable: arcsinh. The dependent variable is the arcsinh of the lost hectares; Deforestation and droughts over the main agricultural periods and cycles; experienced and current, yearly aggregation and by season

	Dependent variable:				
	Arcsinh of deforested hectares				
Droughts:	(1) Experienced & current yearly aggregation	(2) Experienced, detailed	(3) Current, detailed	(4) Current, aggregated	(5) Experienced, aggregated
Yearly aggregation					
Experienced, y	-0.0196				
	(0.0132)				
Current, y	0.0010				
	(0.0127)				
Cassava					
Planting		-0.0246	0.0760***	0.0720**	-0.0250
		(0.0281)	(0.0289)	(0.0287)	(0.0284)
Harvesting		-0.0718	-0.0997*	-0.0701	-0.0634
		(0.0487)	(0.0593)	(0.0611)	(0.0516)
Maize, seasonality detailed					
Planting 1		0.0081	-0.0775***		
		(0.0195)	(0.0220)		
Planting 2		0.0233	-0.0297		
		(0.0193)	(0.0270)		
Growing 1		-0.0555**	-0.0838***		
		(0.0248)	(0.0320)		
Growing 2		0.0037	-0.0632**		
		(0.0233)	(0.0292)		
Harvesting 1		-0.0270	0.1083***		
		(0.0219)	(0.0301)		
Harvesting 2		0.0025	0.0326*		
		(0.0147)	(0.0170)		
Maize, seasonality aggregated					
Planting 1, 2				-0.0363**	0.0171
				(0.0160)	(0.0197)
Growing 1,2				-0.0769***	-0.0302*
				(0.0231)	(0.0171)
Harvesting 1,2				0.0705***	-0.0204
				(0.0152)	(0.0133)
Observations	519,160	519,160	519,160	519,160	519,160
F Statistic	24.1449***	24.0904***	68.9845***	74.2039***	34.9552***
	(df = 2; 493181)	(df = 8; 493175)	(df = 8; 493175)	(df = 5; 493178)	(df = 5; 493178)
Note:					$^{*}p < 0.1$; $^{**}p < 0.05$; $^{***}p < 0.01$
					Time and cell fixed effects, clustered at the sector administrative level

S4 Table. Maize. Deforestation and droughts over maize agricultural seasons. Yearly aggregation, current and experienced, aggregated and by season

	Dependent variable:				
	Log of deforested hectares +1				
Droughts:	(1) Experienced & current yearly aggregation	(2) Experienced, detailed	(3) Current, detailed	(4) Current, aggregated	(5) Experienced, aggregated
Yearly aggregation					
Experienced, y	-0.0168				
	(0.0115)				
Current, y	0.0002				
	(0.0110)				
Seasonality detailed					
Planting 1		0.0082	-0.0592^{***}		
		(0.0170)	(0.0179)		
Planting 2		0.0062	-0.0215		
		(0.0181)	(0.0229)		
Growing 1		-0.0561^{**}	-0.0582^{**}		
		(0.0224)	(0.0277)		
Growing 2		0.0054	-0.0565^{**}		
		(0.0220)	(0.0251)		
Harvesting 1		-0.0392^{**}	0.0909^{***}		
		(0.0195)	(0.0258)		
Harvesting 2		-0.0039	0.0244^{*}		
		(0.0134)	(0.0148)		
Seasonality aggregated					
Planting 1, 2				-0.0243^{*}	0.0145
				(0.0130)	(0.0172)
Growing 1,2				-0.0628^{***}	-0.0300^{**}
				(0.0200)	(0.0153)
Harvesting 1,2				0.0573^{***}	0.0218^{*}
				(0.0133)	(0.0117)
Observations	519,160	519,160	519,160	519,160	519,160
F Statistic	24.1580^{***}	39.4250^{***}	80.9361^{***}	104.8111^{***}	50.4334^{***}
	(df = 2; 493181)	(df = 6; 493177)	(df = 6; 493177)	(df = 3; 493180)	(df = 3; 493180)
Note:					$^{*}p < 0.1$; $^{**}p < 0.05$; $^{***}p < 0.01$
					Time and cell fixed effects, clustered at the sector administrative level

S5 Table. Cassava. Deforestation and droughts over cassava agricultural seasons; Yearly aggregation, current and experienced, by season

	Dependent variable:		
	Log of deforested hectares +1		
Droughts:	(1) Experienced & current yearly aggregation	(2) Experienced, detailed	(3) Current, detailed
Yearly aggregation			
Experienced, y	-0.0368		
	(0.0228)		
Current, y	0.0080		
	(0.0240)		
Seasonality detailed			
Planting		-0.0303	0.0268
		(0.0247)	(0.0243)
Harvesting		-0.0763^{*}	-0.0469
		(0.0451)	(0.0560)
Observations	519,160	519,160	519,160
F Statistic (df = 2; 493181)	28.0392^{***}	38.3600^{***}	8.6174^{***}
Note:			$^{*}p < 0.1$; $^{**}p < 0.05$; $^{***}p < 0.01$
			Time and cell fixed effects, clustered at the sector administrative level

S6 Table. Second Congo War Deforestation and droughts over the main agricultural periods and cycles, excluding the Second Congo War (exclusion of years prior to 2003). Panel 2004-2020; experienced and current, yearly aggregation and by season

	Dependent variable:				
	Log of deforested hectares +1				
Droughts:	(1) Experienced & current yearly aggregation	(2) Experienced, detailed	(3) Current, detailed	(4) Current, aggregated	(5) Experienced, aggregated
Yearly aggregation					
Experienced, y	-0.0235*				
	(0.0126)				
Current, y	-0.0013				
	(0.0117)				
Cassava					
Planting		0.0050	0.0853***	0.0786***	0.0027
		(0.0273)	(0.0256)	(0.0262)	(0.0266)
Harvesting		-0.0635	-0.0961*	-0.0691	-0.0699
		(0.0445)	(0.0529)	(0.0547)	(0.0484)
Maize, seasonality detailed					
Planting 1		-0.0238	-0.0856***		
		(0.0167)	(0.0204)		
Planting 2		0.0440**	-0.0285		
		(0.0195)	(0.0254)		
Growing 1		-0.0789***	−0.0862***		
		(0.0301)	(0.0272)		
Growing 2		0.0179	−0.0482*		
		(0.0211)	(0.0258)		
Harvesting 1		-0.0804***	0.0939***		
		(0.0206)	(0.0260)		
Harvesting 2		-0.0019	0.0401***		
		(0.0159)	(0.0155)		
Maize, seasonality aggregated					
Planting 1,2				-0.0453***	-0.0107
				(0.0150)	(0.0169)
Growing 1,2				-0.0645***	-0.0223
				(0.0203)	(0.0182)
Harvesting 1,2				0.0721***	−0.0291**
				(0.0132)	(0.0132)
Observations	441,286	441,286	441,286	441,286	441,286
F Statistic	40.5554***	69.3159***	84.6469***	92.5989***	44.8462***
	(df = 2; 415310)	(df = 8; 415304)	(df = 8; 415304)	(df = 5; 415307)	(df = 5; 415307)
Note:					$^{*}p < 0.1$; $^{**}p < 0.05$; $^{***}p < 0.01$<br>Time and cell fixed effects, clustered at the sector administrative level
